# Insulinoma diagnosis and characterization with intratumoral heterogeneity employing [^18^F]FB(ePEG12)12-exendin-4 PET/MRI

**DOI:** 10.1007/s00259-025-07513-7

**Published:** 2025-08-21

**Authors:** Hayao Yoshida, Takaaki Murakami, Kanae Kawai Miyake, Yoichi Shimizu, Koji Itagaki, Kentaro Sakaki, Daisuke Otani, Hiroyuki Fujimoto, Daisuke Yabe, Nobuya Inagaki, Yuji Nakamoto

**Affiliations:** 1 Department of Diabetes, Endocrinology, and Nutrition, Graduate School of Medicine, Kyoto University, Kyoto, Japan; 2 Department of Diagnostic Imaging and Nuclear Medicine, Graduate School of Medicine, Kyoto University, Kyoto, Japan; 3 Division of Clinical Radiology Service, Kyoto University Hospital, Kyoto, Japan; 4 Radioisotope Research Center, Agency for Health, Safety and Environment, Kyoto University, Kyoto, Japan; 5 Medical Research Institute KITANO HOSPITAL, PIIF Tazuke-Kofukai, Osaka, Japan; 6 Department of Diabetes, Endocrinology and Nutrition, Kyoto University Graduate School of Medicine, Yoshida-Konoe-cho, Sakyo- ku, Kyoto, 606-8501 Japan

**Keywords:** Insulinoma, Glucagon-like peptide-1 receptor, β-cell imaging, Positron Emission Tomography / Magnetic Resonance Imaging


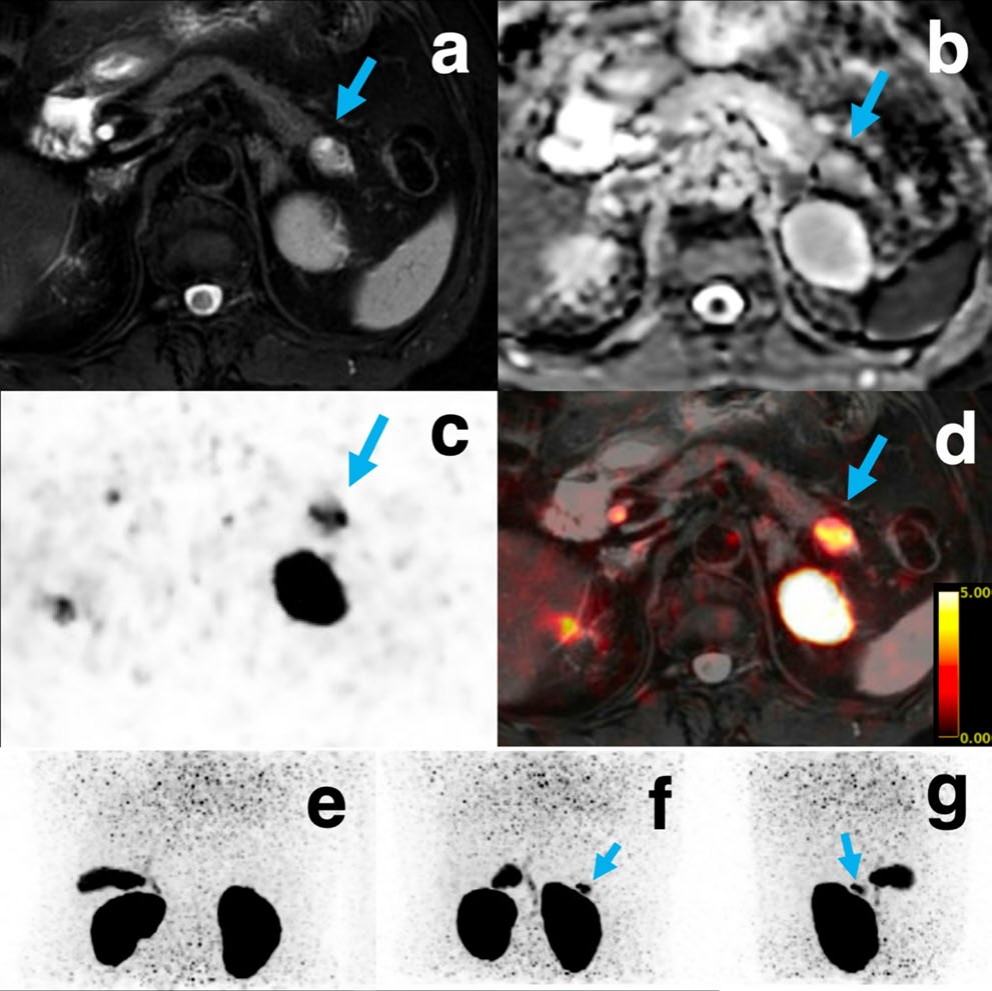


Insulinomas are rare, typically small pancreatic neuroendocrine tumors that often elude detection on widely used imaging methods like computed tomography (CT), magnetic resonance imaging (MRI), and somatostatin receptor (SSTR)-based positron emission tomography (PET), owing to their small size and low levels of SSTR expression [1]. Technically demanding procedures like endoscopic ultrasound and selective arterial calcium stimulation testing can improve sensitivity, but their invasive nature may preclude clinical use [2]. Conversely, because most benign insulinomas display increased levels of glucagon-like peptide-1 receptors (GLP-1R), we developed a PEGylated exendin‑4 probe labeled with [^18^F]FB(ePEG12)12‑exendin‑4 (^18^F-exendin-4)—that enables both qualitative and quantitative diagnosis [3–5].

A 76‑year‑old woman with symptomatic hypoglycemia (plasma glucose 37 mg/dL; C‑peptide 1.87 ng/mL) underwent integrated ^18^F-exendin-4 PET/MRI at the expiratory phase. Figure shows matched panels: (a) fat-saturated T2-weighted MRI and (b) apparent-diffusion-coefficient (ADC) map depict a tumor with a hyperintense cystic component; (c) respiratory-gated PET slice and (d) the fused PET/MRI, (plus (e)–(g), maximum intensity projection for orientation) demonstrates intense uptake confined to the solid, low-ADC core while the cystic component shows negligible activity. After enucleation, hypoglycemia resolved completely and histology confirmed insulinoma.

Owing to its high soft-tissue contrast and motion-corrected spatial resolution, integrated PET/MRI not only delineated the intratumoral characteristics but also clearly distinguished the lesion from adjacent renal physiologic activity—an advantage over sequential PET/CT, which is prone to misregistration and false negatives near the kidney [6, 7]. This case highlights the diagnostic value of GLP-1R-targeted PET/MRI for precise insulinoma characterization and localization.

## Data Availability

Some or all of the datasets generated and/or analyzed during the present study are not publicly accessible. However, they can be obtained from the corresponding author upon reasonable request.

## Declarations

### Clinical trial registration

The study protocol was approved by The Kyoto University Certified Review Board (Y0074, Registered 1 March 2021). The first subject was enrolled on August 12, 2022. All procedures were conducted in accordance with the Declaration of Helsinki and other relevant ethical guidelines. Written informed consent was obtained from the participant prior to their inclusion in the study.
